# Supplementary material for: Subjective feeling of control during fNIRS-based neurofeedback targeting the DL-PFC is related to neural activation determined with short-channel correction
Source: PLoS One. 2023 Aug 16;18(8):e0290005. doi: 10.1371/journal.pone.0290005 (PMC10431651; doi:10.1371/journal.pone.0290005)
Supplement: S1 Table — The mental strategies were acquired by verbal report (interview) at the end of the neurofeedback session in front of two experimenters. These reports were anonymized and then the strategies were classified by the experimenters and approved by another experimenter who was not present during the neurofeedback sessions. Strategies reported as "Cheering" are related to self-encouragement and/or gauge encouragement. “Visualization” strategies are related to visualizing a specific situation or object (including memory recall). “Concentration” strategies are related to any strategy that requires specific mental effort beyond visualization or memory recall. “Emotion” strategies are related to any strategy that was specifically focused on an internal feeling. Because some participants tried different strategies, the strategy that was applied more frequently and/or related to the participants’ internal sense of control over the gauge was selected for analysis. Indeed, when a strategy appeared to be more effective for controlling the gauge, it was the strategy used by the participant most of the time. (DOCX) [file pone.0290005.s002.docx]

| Participant | Gender | Strategies reported (interview recorded) | Classification(s) | Main strategies used for analysis | Feeling control of the gauge (y/n) |
| --- | --- | --- | --- | --- | --- |
| 1 | F | Visualization (ineffective), better with concentration on a specific activity (painting) and positive thoughts toward the gauge | Visualization, concentration | visualization | Y |
| 2 | F | Encouraging the gauge, repetition of positive sentences toward it, wasn't thinking on specific things | Cheering | Cheering | N |
| 3 | M | Encouraging the gauge to increase, was looking at the top of the bar | Cheering | Cheering | Y |
| 4 | F | Concentration on the gauge, which led to a drop of its level, concentration and association with an image was more effective | Concentration, visualization | Concentration | N |
| 5 | M | Encouraging the gauge, was thinking "rise up" and looked at the top of the bar | Cheering | Cheering | Y |
| 6 | M | Planification of a to-do list (not very effective), motivation or wanting something was more effective | Visualization | Visualization | N |
| 7 | M | Visualization of movements of its body, encouraging the gauge, was focused on color orange/red (top of the gauge) | Visualization, cheering | Visualization | Y |
| 8 | M | Being angry toward the gauge more effective rather than having positive thoughts | Emotion | Emotion | N |
| 9 | M | Mental arithmetic | Concentration | Concentration | N |
| 10 | F | Thinking at positive memories was more effective rather than negatives memories, mental arithmetic | Visualization, concentration | Visualization | N |
| 11 | M | Muscle contraction, feeling positive when the gauge was rising, think of "being happy" when gauge become red, focalized on PFC brain region | Concentration, motor | Concentration | N |
| 12 | F | Was thinking on the discussion part of her article (felt more effective on NF success), thinking of a work to-do-list, planification (was less effective) | Concentration | Concentration | N |
| 13 | F | Encouraging the gauge (saying "rise up"), association with a loading file | Cheering and visualization | Cheering | Y |
| 14 | F | Was concentrated (looked at the top of the bar, wanted to increase the color's bar to the red), and encouraging the gauge | Concentration, cheering | Concentration | Y |
| 15 | M | Meditation/relaxation, visualization of a well-being place with auditory noise (water) | Relaxation, visualization | Visualization | Y |
| 16 | F | Thinking of specific (important) decisions to do, concentration on to do list | Concentration | Concentration | Y |
| 17 | M | Imagination of feeling emotion (stress/panic more efficient than joy) but don't know if it was due to the increased respiration rate | Emotion | Emotion | N |
| 18 | M | Thinking at lots of things, mental arithmetic, repetition of a sentence, synchronization of respiration during one NF trial | Concentration, respiration | Concentration | N |
| 19 | F | "Telekinesis" to increase the gauge, encouraging the gauge, ocular movements | Concentration, cheering, motor | Cheering | Y |
| 20 | F | Concentration on the gauge | Concentration | Concentration | Y |
| 21 | M | Questioned himself to take decisions, concentration | Concentration | Concentration | N |
| 22 | F | Increase the gauge with eyes, respiration, focus on beeing relaxed, focused/being aware of its respiration (without changing it) | Concentration, motor | Concentration | N |
| 23 | F | Thinking/visualize at something motivating (boxing) | Visualization | Visualization | N |
| 24 | M | Visualization of the gauge at the maximum, looked at the top of the bar | Visualization | Visualization | Y |
| 25 | F | Visualization of positive memories | Visualization | Visualization | Y |
| 26 | M | Encouraging the gauge to increase | Cheering | Cheering | N |
| 27 | F | Concentration on specific task and on the gauge | Concentration | Concentration | Y |
| 28 | M | Encouraging the gauge, self-encouragement | Cheering | Cheering | Y |
| 29 | M | Encouraging the gauge | Cheering | Cheering | Y |
| 30 | F | Concentration on specific tasks | Concentration | Concentration | N |

**Supplementary Table 1**

The mental strategies were acquired by verbal report (interview) at the end of the neurofeedback session in front of two experimenters. These reports were anonymized and then the strategies were classified by the experimenters and approved by another experimenter who was not present during the neurofeedback sessions. Strategies reported as "Cheering" are related to self-encouragement and/or gauge encouragement. Visualization strategies are related to visualizing a specific situation or object (including memory recall). Concentration strategies are related to any strategy that requires specific mental effort beyond visualization or memory recall. Emotion strategies are related to any strategy that was specifically focused on an internal feeling. Because some participants tried different strategies, the strategy that was most applied and/or related to their internal sense of control over the gauge was selected for analysis. Indeed, when a strategy seemed to be more effective for controlling the gauge, it was the strategy most used by the participant.
