# Supplementary material for: Subjective feeling of control during fNIRS-based neurofeedback targeting the DL-PFC is related to neural activation determined with short-channel correction
Source: PLoS One. 2023 Aug 16;18(8):e0290005. doi: 10.1371/journal.pone.0290005 (PMC10431651; doi:10.1371/journal.pone.0290005)
Supplement: Attachment — Submitted filename: PLoS_ReviewerComments_NF_fNIRS_16May23.docx [file pone.0290005.s004.docx]

Summary of Article:

One goal of this manuscript was to provide results from a study which examined differences between two separate band-pass filters ranges on the quality of fNIRs measures of hemoglobin based physiological mechanisms underlying brain activity during a 15-trial neurofeedback session. The study further aimed to examine the methodological advantages of including short channel signal as a regressor in their brain activity analysis models with the goal of reducing non-brain related signal during a neurofeedback session. In addition, the study assessed the degree to which individual’s self-reported mental strategies and subjective feelings of control during the neurofeedback session were associated with their brain activity.

Major Points:

Overall, this manuscript was well written and interesting, and the findings from this study provide valuable insights and information for both researchers and clinicians utilizing neurofeedback and fNIRS neuroscience methodologies. With a few adjustments I think this paper would be a valuable contribution to the field of neuroscience research, specifically for research involving neurofeedback. I think this manuscript would make a strong contribution to the aims of *PloS One* if the following concerns are addressed. I also want to preface my reviewer comments by saying that I am not an expert in the field of fNIRS or neurofeedback, though I have some experience in this area. I am more well-versed in the cognitive EEG literature. However, I believe if my reviews are addressed, this will help the manuscript be more accessible for a broader audience of readers, which is a goal of *PloS One*.

A primary concern or area for improvement for this manuscript is a seeming lack of introductory information to set the stage for a broader reader base to better understand the study.

1. For one, I would like to see more info that describes the rationale for choosing the specific band-pass filters employed in the current study. I would like to see more background information on what filters prior studies have used and what limitations they encountered.
2. Similarly, I would like to see more description of background information on the prior use or non-use, limitations, and strengths of different SC corrections.
3. In addition, it would be helpful to provide more description of the NF methodologies employed, specifically regarding the choice and purpose of the sources and detectors.

Another major concern is the apparent lack of complete statistical values for the analyses. I would like to see beta weights, t-values, p-values, and effect sizes if possible. This is important not only for meta-analyses but also for future work that will utilize this study to inform their research using fNIRS and NF.

Another major concern I had with reading the methods/results section is not having a clear understanding of which electrodes were utilized in the analyses. Is it possible to provide channel labels or some way to identify specifically which cortical locations were used for analysis?

Minor points:

The following points are suggestions for areas for improvement of this paper to improve broader reader comprehension and field specific understanding of the methodological approaches. Keep in mind these are all suggestions as I am not an fNIRS or NF expert. Also, suggestions for various minor grammar/typo/English language issues will be provided as a tracked changes marked-up word doc of the manuscript.

Overall:

1. I suggest sending this manuscript to a writing studio for revision after you make any edits to help with any English language translations issues.
2. Consider exploring current APA 7 style heading formatting.

Paragraph 1 of intro:

1. Run-on sentence. Consider Revising.
   a. “Although the temporal resolution is lower than the resolution achieved by EEG, and only cortical brain activity can be observed, fNIRS appears as to be a good compromise for NF studies and has been increasingly used.”

Paragraph 2 of intro:

1. Does this mean that the research/clinicians will not share their methodology? If not, what is the reason most NF studies do not use SC corrections?
   a. “The use of this approach in fNIRS-based NF studies remains confidential”

Paragraph 4 of intro:

1. Consider providing examples of these strategies.
   a. “Moreover, the degree of awareness understanding of the NF training paradigm, the instructions provided (explicit or implicit) and the type of mental strategy used in order to control the region of interest may profoundly affect NF success”
2. In addition, consider describing the differences between mental strategies, in particular, those explored in the current study.

Paragraph 5 of intro:

1. Consider indicating what the different filter conditions are.
2. Are you making any specific predictions/hypotheses, if so, considering including them here.

Paragraph 2 of methods:

1. Are you basing this sample size from prior work? If so, consider including this info.
2. Consider including a figure image below this paragraph or in supplementary material if possible. Figure 4 should be labeled Figure 1.
3. Also consider including if more mental effort increased or decreased the position of the gauge.

Paragraph 4 of methods:

1. Is it possible to rotate figure 1A to the right and down, and include an XYZ axis? Not necessary, per se, but could be helpful for comprehension. Also, it could be useful to label your electrodes according to the 10-20 system or another channel naming convention.
2. Consider briefly explaining what increases and decreases in oxy-Hb mean in terms of cognitive function.

Paragraph 5 of methods:

1. Consider moving this to earlier in the manuscript. Perhaps at the end of the participants and procedure section.
   a. “See figure 4 for an overview of the protocol design and scheme of NF sessions.”
2. Again I think Figure 4 should be mentioned sooner and thus changed to Figure 1.

Paragraph 6 of methods:

1. Could you provide information about the specifics of the computer monitor? The model, resolution, refresh rate, the distance participants sat from the monitor? If someone were to try and replicate this study to get the same results, what exactly would they need to do to recreate the protocol conditions?

Paragraph 7 of methods:

1. Can you provide an example? What is a to-low filter range? Same for too-high filter?
   a. “a too-low low-pass filter might remove too much relevant neural signal, but a too- high low-pass filter”
2. This is a complicated model for broader audiences to grasp. It could help to include a figure that depicts your models used. You could then refer to that figure in future sections.
3. I’m not familiar with this type of filter. Is it commonly utilized in the NF field? If not, consider providing more information.
   a. “via pre-whitening filters”

Paragraph 8 of methods:

1. When you say “around”, was there a range? Is it important to clarify this?
   a. “was around 30 mm for a long channel and 8 mm for a short-channel”
2. Consider elaborating on this point in the intro as it seems to be a primary goal of your project.
   a. Indeed, the distance for short-channels classically ranges between 0.5 and 1.5 cm [42], but a distance under 10 mm is recommended [43].
3. Is it possible to provide the electrode channel labels according to the 10-20 system? E.g. Fz Cz F3
   a. “Three regions of interest”
4. This was difficult to read and comprehend. Is it possible to rewrite this? Also, is this a common practice for NF research? Can you cite exemplars?
   a. “Given that within a single NF session, each participant was possibly exploring different strategies, not all NF trials were expected to yield successful NF control. We thus decided to investigate the individual channel response in relation with the entire 15 trials, but also the 10 and 5 best trials, determined as the best averaged t-value in the four NF channels computed for each trial.”

Paragraph 8 of methods:

1. Can you indicate which figure numbers here. You are referencing?
   a. “detailed in the figures’ legends”
2. Which figure? Include figure number?
   a. “the figures’ details.”

Subheading 1 of results:

1. Consider replacing with a broader, more general results heading titles.
   a. “One single DL-PFC-targeted NF session promotes effective DL-
   b. PFC activation for at least 10 trials within 15.

Paragraph 1 of results:

1. Do you have tables to reference with the statistical outputs? The B values, t-values, p-values, effect sizes? This would be useful for multiple reasons, especially for meta-analyses.
2. Did you elaborate your predictions in the intro? If not, it would be helpful to do so for the reader to understand what you were measuring.
   a. “we expected an increase in HbO2 and a decrease in HbR”

Paragraph 2 of results:

1. Not sure which channels these are. Consider providing channel labels on figures and in-text.
   a. “four NF channels”
2. Here would be a good place to include the findings for including short-channel correction models similar to above.
   a. “without SC correction”

Subheading 2 of results:

1. Consider replacing with more general results section title.
   a. “Depending on the BP filtering characteristic, the activation within the NF channels is moderately impacted by physiological noise and is not associated with other frontal and motor brain regions activation.”

Paragraph 7 of results:

1. Both this paragraph and the one prior could benefit comprehension if there was an accompanying table that indicated the results. For me, the figures are not intuitive to navigate. Also, that table could include regression beta’s, t’s, and p values. Effects sizes would also be great.
   a. “For the BP 0.01-0.2 Hz filter”

Paragraph 8 of results:

1. It is not clear to me at this point why the narrower band pass filter is better. Consider providing a brief explanation of why this is prior to this sentence.

Paragraph 9 of results:

1. I don’t quite understand what you are comparing for significance here. Is it the number of participants with activation in at least one source/detector value compare participants with 0? Why are you comparing these? Are you trying to demonstrate that when SC are regressed out when using the .01-.20 BP filter that there are significantly more participants showing activation? Does that contradict the statement in the earlier paragraph that says the .01-.09 BP filter is better?
   a. “Chi-square test for the proportion of the participants with activated channel in function of DL-PFC+SC activation or not, * p<0.05.”

Paragraph 10 of results:

1. I’m not sure if this way of phrasing that you are regressing out SC’s is intuitive. Consider stating “for SC regressed models”.
   a. “DL-PFC+SC”
2. All p-values should be reported. Consider making a table for this result section similar to the ones above.
3. Why is this data not shown? Consider including in a table.
   a. “(data not shown).”

Paragraph 11 of results:

1. This section could be elaborated upon with more detail.

Paragraph 1 of discussion:

2. After this sentence, you should provide a brief description of the study aims and procedures.
   a. “In the present study, 30 individuals performed a single session of fNIRS-based neurofeedback (NF) targeting the DL-PFC bilaterally that consisted in of 15 trials of NF alternating with 30-sec alternating resting periods.”
