# Supplementary material for: Subjective feeling of control during fNIRS-based neurofeedback targeting the DL-PFC is related to neural activation determined with short-channel correction
Source: PLoS One. 2023 Aug 16;18(8):e0290005. doi: 10.1371/journal.pone.0290005 (PMC10431651; doi:10.1371/journal.pone.0290005)
Supplement: Attachment — Submitted filename: PlosFNIRsReview_Upshaw_16May23.docx [file pone.0290005.s005.docx]

Neurofeedback (NF) training is a promising preventive and therapeutic approach for brain and behavioral impairments, the dorsolateral prefrontal cortex (DL-PFC) being a relevant region of interest. Functional near-infrared spectroscopy (fNIRS) has recently been applied in NF training. However, this approach is highly sensitive to extra-cerebral vascularization, which could bias measurements of cortical activity. Here, we examined the feasibility of a NF training targeting the DL-PFC and its specificity by assessing the impact of physiological confounds on NF success via short-channel offline correction under different signal filtering conditions. We also explored whether the individual mental strategies affect the NF success. Thirty volunteers participated in a single 15-trial NF session in which they had to increase the oxy-hemoglobin (HbO2) level of their bilateral DL-PFC. We found that 0.01-0.09 Hz band-pass filtering was more suited than the 0.01-0.2 Hz filter to highlight brain activation restricted to the NF channels in the DL-PFC. Retaining the 10 out of 15 best trials, we found that 18 participants (60%) managed to control their DL-PFC. This number dropped to 13 (43%) with short-channel correction. Half of the participants reported a positive subjective feeling of control, and the “cheering” strategy appeared to be more effective in men (p < 0.05). Our results showed successful DL-PFC fNIRS-NF in a single session and highlighted the value of accounting for extra cortical signals, which can profoundly affect the success and specificity of NF training.

Introduction

Neurofeedback (NF) is a neurocognitive training procedure aiming to assist people in learning to self- regulate brain activity in a specific neural region [1]. With the help of neuroimaging techniques (e.g. electroencephalography – EEG, functional magnetic resonance imaging fMRI, functional near-infrared spectroscopy – fNIRS), the activity of a specific brain area can be measured in real-time. During NF, an indicator of this brain activity can be fed back to the participant as a metaphorical representation through a sensory modality (e.g. visual, auditory). The application of NF-based training protocols covers a wide range of research areas, from enhancing cognitive abilities in healthy individuals, to improving health conditions and behaviors in the context of neurocognitive disorders. The NIRS-based NF approach is an alternative to EEG and fMRI with great potential for translational research and clinical applications [2,3]. As an optical brain imaging technique, fNIRS is less expensive and more accessible than fMRI and less sensitive to movement artefacts compared to EEG, leading to a wider range of application in psychiatry and behavioral research among others [4,5]. Although the temporal resolution is lower than the resolution achieved by EEG, and only cortical brain activity can be observed, fNIRS appears to be a good compromise for NF studies and has een increased use. In particular, fNIRS-NF training protocols have been utilized to improve cognitive function in elderly samples [6], in impulsive adults [7], as well as in children and adults diagnosed with attention deficit hyperactivity disorder (ADHD) [8–10]. FNIRS-NF can also be applied to improve motor rehabilitation outcomes in stroke survivors [11]. Beyond specific pathologies, fNIRS-NF has been implemented to improve cognitive abilities (executive functioning), such as working memory [12], emotion regulation [13], and cognitive flexibility [14]. Greater brain activity requires increased oxygen consumption and higher cerebral blood flow. With NIRS, brain activity is measured indirectly by sending continuous light in red and near infrared wavelengths to assess changes in oxy-hemoglobin (HbO2) and deoxy- hemoglobin (HbR) relative concentrations in human tissues [15,16]. That said, one of the drawbacks of the NIRS is related to the measure of unspecific blood flow variations that can originate from systemic physiological changes [2,15,17]. Indeed, vascularization of non- cortical human tissues, such as skin and skull, can also be detected via NIRS. Beyond tissue vascularization, other physiological processes (e.g. cardiac rhythm, respiratory rhythm) can also modify HbO2 concentration. In order to distinguish cortical brain activity from other physiological changes (e.g. cardiac rhythm, variations in arterial blood pressure called Mayer waves, breathing), low band-pass filtering is classically used to remove confounding factors from the NIRS signal [15]. However, discrepancies remain regarding which filter characteristics to use to investigate brain signal activity without removing too much of the brain signal of interest [18,19]. Indeed, for fNIRS-based NF purposes, in which only intermittent neural activation occurs during a NF trial, the filtering option used for other fNIRS applications might not be appropriate, such as for evoked-related activation analysis [cite?]. To handle the contamination of extra-cerebral tissue signals in particular, the use of short-channels (SC) is encouraged [20,21]. By reducing the distance between the light sources and detectors, the depth of the light pathway is reduced, leading to a measure of extra-cerebral tissues signal only. Thus, the SC signals can be used as regressor for subsequent offline statistical analysis. The use of this approach in fNIRS-based NF studies remains confidential [22,23] and to date, most of fNIRS-NF studies have been conducted without SC. Since the fNIRS-NF setup without SC might be better suited for clinical purpose, it is thus important to investigate the link between online NF signal without SC, experienced by the participants, and the offline SC-corrected signal, which can be more related to proper neuronal activation. Recently, some efforts have been made to unify the practices for fNIRS data acquisition and analysis [19].

Different regions of interest can be targeted in NF training protocols. The dorsolateral prefrontal cortex (DL-PFC) is a common target to train cognitive processing [7,9,10,12,13,24– 28]. Some studies have shown that individuals are better at controlling DL-PFC activity in NF training [9,12,13], but these finding remain scarce and more research is needed to confirm its efficacy. To our knowledge, only one study has used a SC regression during post-processing in an fNIRS-based NF training targeting the DL-PFC [10]. A separate study used SC to integrate them during the feedback, but with 1.5-mm distance between the SC [29]. Thus, there is a need to further investigate whether and how scalp vascularization may affect the NF success before its implementation in a clinical context.

Beyond the confounding factors related to measurement for fNIRS-NF in particular, another limitation of NF in general is the fact that not all subjects respond. Indeed, NF success depends on the participants’ motivation, involvement in the training and learning capabilities [30,31]. These factors can contribute to a range of 15 – 30 percent of individuals who are unable to self-monitor their brain activity at the end of an NF training protocol [1,32]. These individuals are classically referred to as “non-responders” compared to “responders” who were successful in the task. Moreover, the degree of understanding of the NF training paradigm, the instructions provided (explicit or implicit) and the type of mental strategy used to control the region of interest may profoundly affect NF success [33,34]. In accordance with the CRED-nf checklist [35], investigating the mental strategies used by an individual is highly recommended, as they are related to NF learning success and should be reported in NF training protocols.

The purpose of this fNIRS-based NF targeting the DL-PFC study is to (i) assess whether individuals are able to voluntarily and specifically increase their bilateral DL-PFC activity across a single NF session, (ii) assess the specificity of cortical DL-PFC activation during NF with offline SC correction and under different signal filtering conditions, and (iii) explore the mental strategy used in relation to the NF success.

Materials and Methods Ethics statement

This study protocol was approved by the ethics committee of Rennes University-Hospital (“Comité d’éthique du CHU Rennes”, n°21.128), and was conducted in accordance with the French and European General Data Protection Regulation 2016/679 and the Declaration of Helsinki. Free informed consent was obtained from all participants. The data used and provided in this paper or as supplemental data were completely anonymized, with no means to infer the identity of participants.

Participants and study procedure

Thirty participants, between 19 and 60 years old (*M* (*SD)*= 38 (12) years old), female/male ratio=1/1, were recruited. As we aimed to explore our neurofeedback (NF) approach and set-up, we expected that 30 participants should be enough for this validation study. They were asked to perform a single functional near-infrared spectroscopy (fNIRS) NF session consisting of 15 trials in which participants were asked to increase the level of a gauge. As recommend by Strehl et al. (2014), no explicit strategy was imposed nor suggested, letting the participants identify by themselves the mental strategy that suited them best [31]. Participants were simply informed that the level of the gauge was a metaphor correlated with the activity of the dorsolateral prefrontal cortex (DL-PFC), a brain region involved in goal-oriented behaviors, cognitive and self-control [5]. This kind of neurofeedback task is classified as explicit, as the individuals were aware of the meaning of the gauge signal and knew which brain region was targeted. On the contrary, the provided task instructions are classified as implicit as no explicit strategy was provided [33].

Behavioral assessments

In accordance with the CRED-nf checklist [35], the subjective feeling of success and the mental strategies used to control the gauge were gathered through an interview after the NF session. The participants perception of controlling the gauge for the entire session was recorded as a binary “yes”/“no” response a posteriori. The participants’ *best* strategy for controlling the gauge was also recorded and then classified by the experimenters according to Kober et al. (2013), which included visualizing, cheering, concentration, emotion [34] or anger as an additional strategy. A complete report of behavioral assessment is presented in Supplementary Table 1.

Functional near-infrared spectroscopy (fNIRS) neurofeedback

fNIRS signals were acquired with an 8 LED sources (λ1|2 = 760|850 nm) / 8 APD detectors NIRScout XP (NIRX, Berlin, Germany) system (sample rate = 7.8125Hz) coupled with the NIRStar software (v15.2, NIRx, Berlin, Germany) and Lab Streaming Layer LSL (San Diego, USA, https://zenodo.org/record/6387090). The oxy-hemoglobin signal, converted to concentration changes (in μM) using the modified Beer-Lambert law by NIRStar from raw signal, is considered to reflect cortical activation variations and was used for real-time feedback calculation with a dedicated software implemented in Python. Psychopy was used for feedback presentation to the participant [36]. As presented in Figure 1A and 2A, sixteen channels were dedicated to record the frontal region, one channel was located on the motor region and one detector was used for short-channel measurement of all channels, i.e. superficial signal from the scalp (Figure 2A). For the feedback calculation and presentation, only the four channels covering bilaterally the DL-PFC were considered as previously described [37] (Figure 1A). We used a moving average of 2 sec, and a band-pass filter (Butterworth, order 3, 0.01- 0.2 Hz) [18,19]. The mean value of these four channels was computed and subtracted by the mean signal value of the 5 seconds preceding the NF task to account for signal baseline. The visual feedback consisted in a simple gauge related to the online computed signal. The gauge is made of steps from yellow (bottom) to red (top), half for positive changes in concentration and half for negative changes. If there were no changes in concentration, the participant sees the gauge at half height. If oxy-Hb increases, the gauge will increase and if oxy-Hb decreases, the gauge will decrease. At both ends, the gauge is not linear to avoid “saturation” effects.

The NF session consisted of a two-minute rest, followed by 15 repetitions of: NF task (30 sec) and rest (30 sec). During rest, a concentric blue circle alternatively increasing and decreasing in diameter (0.1 Hz, fixed duration, not related to measured signals) was presented to help participants disengage from the ongoing cognitive strategy developed during the NF task. See figure 4 for an overview of the protocol design and scheme of NF sessions. A CRED-nf check list [35] is provided in Supplementary Table 2.

Figure 4. Experimental design and installation of the participant during real-time Neurofeedback training. A. Protocol design. Each NF session consisted in 15 trials of 30 seconds of NF with the colored gauge, alternating with 15 rest periods with a moving blue circle to help them to disengage from the task. During the baseline period, the blue circle also appeared on the computer screen of the participant. B. Layout of the NIRS cap. C. Schematics of the installation of the participant in a dark-dedicated room. The experimenter was hidden behind the participant’s computer screen. The NIRScout system was connected to the experimenter’s computer to launch the software used for data acquisition and signal feedback. During rest periods, the experimenter could still see the gauge and raw signals, as well as cardiac and breathing rhythms. The task was computed and launched through ©Psychopy and extended on the computer screen of the participant.

fNIRS-based activation analysis

Activation maps were obtained with the NIRS toolbox [38]. The raw data were converted into optical density, then bandpass-filtered (BP filter: Either 0.01Hz to 0.09Hz or 0.01Hz to 0.2Hz) to remove possible confounding signal such as heartbeat, breathing and Mayer waves. We chose to investigate two signal filtering options given that no consensus currently exists and a too-low low-pass filter might remove too much relevant neural signal, but a too-high low-pass filter might conversely keep too much non relevant physiological confounding non-brain signal. The signal was then resampled (native Matlab resample function) at a rate of 4 Hz before being converted into oxy- and deoxy-hemoglobin by the modified Beer-Lambert law [39]. A generalized linear model (GLM) based on auto-regressive iterative reweighted least squares analysis was used to model the NF task-related hemodynamic response at the individual level [40]. NF task and rest were added to the GLM model and the NF task onsets were used for activation analysis. This model enables via pre-whitening filters to correct serial correlations and outliers caused by motion artifacts. HRF was modeled using the default canonical one from the NIRS toolbox (peak time = 4s, under shoot time = 16s, duration = 32s, ratio = 1/6). Short channel signals were added to the GLM model and eventually used as regressors in order to remove physiological oxy-hemoglobin signal variations from the scalp, which can result from a type of biofeedback (e.g. voluntary or involuntary control of heart rate).

The standard distance between sources and detectors was around 30 mm for a long channel and 8 mm for a short-channel , which is the optimal distance in order to only detect non-cerebral NIRS activation for the frontal lobe [41]. Indeed, the distance for short-channels classically ranges between 0.5 and 1.5 cm [42], but a distance under 10 mm is recommended [43]. For each channel and for each of the 15 trials, beta and t-values were computed. Three regions of interest were analyzed (i) DL-PFC NF region (4 channels), (ii) other frontal brain regions (12 channels), and (iii) motor brain region (1 channel). Based on the number of channels in each studied brain region, a Bonferroni correction for multiple comparison was applied separately for each region of interest. For each participant and brain region of interest, we then computed the number of activated channels as defined by a positive estimator beta-value reaching a Bonferroni corrected statistical threshold of pBonferroni < 0.05. Given that within a single NF session, each participant was possibly exploring different strategies, not all NF trials were expected to yield successful NF control. We thus decided to investigate the individual channel response in relation with the entire 15 trials, but also the 10 and 5 best trials, determined as the best averaged t-value in the four NF channels computed for each trial.

Statistical analysis

Chi-square tests were used with SPSS software (SPSS, Chicago, Illinois, United States) for group comparisons as detailed in the figures’ legends. A p-value < 0.05 was considered significant. A person blind to the NF data acquisition session information performed statistical analyses. As n exploratory approach, we also investigated the effect of gender effect in some analyses as seen in the figures’ details.

Results

One single DL-PFC-targeted NF session promotes effective DL-

PFC activation for at least 10 trials within 15.

We performed a GLM statistical analysis on each of the four channels used for feedback calculation and presentation during the 15 NF trials (Figure 1A). Although the results are based on the GLM statistical analysis, an example of a block-average is depicted in Figure 1B. According to the blood oxygen level dependent (BOLD) hemodynamic response function (HRF), we expected an increase in HbO2 and a decrease in HbR during the NF task, reflecting increased brain activity due to the metabolic demand. In contrast, we expected no relative changes during rest periods, because we did not expect any specific involvement of the DL-PFC then (Figure 1B). Note that the two BP filters promoted similar block average results, even if BP 0.01-0.2 Hz show a more oscillating signal.

With the BP 0.01-0.09 Hz filtering, when performing the analysis on all 15 trials, two participants had a statistically significant increased activity in at least one NF channel without SC correction (DL-PFC) and twelve with SC correction (DL-PFC+SC, Figure 1C). Since NF requires training and the participants can explore various strategies to increase the gauge, we cannot systematically expect all 15 NF trials to be successful. Consequently, we also performed an analysis on a selection of the 10 and 5 best trials corresponding to the average of the 10 and 5 best t-value over the four NF channels within the 15 trials for each participant. When considering the 10 best trials, eighteen (60%) participants had increased activity in at least one NF channel without SC correction. For the 5 best trials, there was no difference in the number of participants with at least one activated channel without SC correction (N = 18).

With the BP 0.01-0.2Hz filtering, when performing the analysis on all 15 trials, twelve participants had significantly increased activity in at least one NF channel without SC correction (DL-PFC) and sixteen participants with SC correction (DL-PFC+SC, Figure 1D). When considering only the 10 best trials, the number of participants that had an increased activity in at least one NF channel significantly increased to 25 (83%) without SC correction. The selection of the 5 best trials did not result in an improvement in the number of participants with at least one activated channel without SC correction (N=27). For further analysis, we investigated the channel activation for the 10 best NF trials at the individual level.

Figure 1. Number of activated channels as a function of the best-selected trials of the 30 participants (15-all, 10, or 5) and two different BP filters. A. Frontal view of the functional near-infrared spectroscopy (fNIRS) layout with the region of interest (ROI) neurofeedback (NF) channels (n = 4) used for feedback calculation covering the dorsolateral prefrontal cortex (DLPFC, green). The layout consisted of a coupling of eight sources and seven detectors; Detector 8 was used to collect the short-channel (SC) signal for all sources (purple circle). The respective locations of sources (red) and detectors (blue) are indicated. B. Block-average on activated channels within the NF channels from an individual who succeeded in NF training. Red lines represent mean HbO2 concentration and blue lines represent mean HbR concentration. See Supplementary Figure 1 for the block-average over the 30 individuals for the average signal in the four DL-PFC NF channels for the 10 best trials. Distribution of the participants according to the number of activated channels within the four NF channels for the entire 15 trials, or the 10 and 5 best trials respectively for C: BP 0.01-0.09Hz filter and D: BP 0.01-0.2Hz filter. Chi-square comparison of the proportion of participants with at least one activated channel for the 10 and 5 best trials vs 15-all trials, * p<0.05.

Depending on the BP filtering characteristic, the activation within the NF channels is moderately impacted by physiological noise and is not associated with other frontal and motor brain regions activation.

To remove scalp generated physiological noise we performed additional GLM analyses in which we regressed out the signal from the short-channel (SC) detectors located on each source channel (Figure 2A) (DL- PFC+SC).

For the BP 0.01-0.09 Hz filter, 13 out of 30 participants( 43%) were identified as having at least one of four activated NF channels with the DL-PFC+SC analysis (Figure 2B). Differences were found between the DL-PFC+SC and the DL-PFC analyses (Figure 2B). Three participants who did not present at least one activated channel with the DL-PFC analysis presented an activated channel with the DL-PFC+SC analysis (Figure 2C). Conversely, eight participants with at least one activated channel with the DL-PFC analysis were not detected with the DL-PFC+SC analysis. Overall, both analyses concurred with the detection of 10 participants among 30(33%) for whom at least one channel was activated within the four channels used for NF. With the SC regression, the activation in at least one of the four NF channels did not reveal any relevant change in the pattern of channel activation in the other frontal brain regions (Other frontal+SC, Figure 2B and C) nor in the motor brain area (Motor+SC, Figure 2B and C).

For the BP 0.01-0.2 Hz filter overall, , 21 out of 30 participants( 70%) were identified as having at least one of the four activated NF channels with the DL-PFC+SC analysis (Figure 2B). The DL-PFC+SC and the DL-PFC analyses yielded some differences (Figure 2B). Three participants who did not present at least one activated channel with the DL-PFC analysis presented an activated channel with DL-PFC+SC analysis (Figure 2C). Conversely, seven participants who were identified as having at least one activated channel with the DL-PFC analysis were not detected with the DL- PFC+SC analysis. Overall, both analyses concurred with the detection of 18 participants among 30 (60%) for whom at least one channel was activated within the four channels used for NF. With the SC regression, the activation in at least one of the four NF channels was associated with the activation in at least one of the twelve other frontal channels (Other frontal+SC, Figure 2B and C), and the motor channel (Motor+SC, Figure 2B and C).

As we found only a selective activation of channels in the DL-PFC without an association with the activation in other frontal and motor channels for the BP 0.01-0.09Hz filter, we further analyzed participants’ subjective feeling of controlling the gauge with this BP filter, as it might highlight a more reliable brain activation related to NF.

Figure 2. Characterization of the neurofeedback (NF) signal. A. Frontal view of the functional near-infrared spectroscopy (fNIRS) layout with (i) the NF channels (n = 4) used for feedback presentation covering the DL-PFC (green), (ii) other frontal channels (n=12) covering the frontal area (brown), and (iii) one channel covering the motor area in the temporal lobe (black). The layout consisted in a coupling of eight sources and seven detectors; detector 8 was used to collect the short-channel (SC) signal for all sources (purple circle). The respective locations of sources (red) and detectors (blue) are indicated. B. For each BP filtering and for each volunteer, number of activated channels within the four NF channels with (DL-PFC+SC) and without (DL-PFC) SC regression (i.e. exclusion or inclusion of the scalp physiological signal, respectively), as well as the number of activated channels in other frontal (Other frontal+SC) and motor areas (Motor+SC) with SC regression. C. For each BP filtering, influence of the scalp physiological signal on the NF channels activation (comparison of subjects identified as having 0 or at least ≥ 1 activated channel between the DL-PFC+SC and the DL-PFC analyses, i.e. with and without SC regression). Proportions of subjects with activation in other frontal regions and motor area in those categorized as having 0 or at least 1 activated channel with the DL-PFC+SC analysis (with SC regression for all). The numbers of subjects for each condition are indicated in the pie charts. Chi-square test for the proportion of the participants with activated channel in function of DL-PFC+SC activation or not, * *p* < 0.05.

NF feeling of control is related with higher NF channels activation without preferential mental strategy

Among the thirty participants, half reported a positive control feeling while the other half did not experience the feeling of controlling the gauge (Figure 3A). With the DL-PFC+SC analysis of activated channels, we detected a non-significant trend toward a higher number of participants with at least one activated channel within the NF channels when participants reported a feeling of control (Control vs. No Control, *p* = 0.065). Among those who reported a feeling of control, we detected at least one activated channel NF channel in 9 participants,(60%). For participants that did not report a feeling of control, only four participants, (27%), presented an activated NF channel. Note that the analysis between control feelings and DL-PFC (without SC) provided similar results (data not shown). Interestingly, the difference in activated NF channels related to the feeling of control was more pronounced in women than in men (p < 0.05), i.e. men reported feeling less control although an activation was still observed.

Even though we only detected a statistical difference in men for the mental strategy used between the groups of participants, i.e. with or without gauge control feeling, (Figure 3B), the “cheer” strategy appeared to be more associated with a control feeling. Additionally, we did not detect a preferential strategy related to DL-PFC+SC channel activation.

Figure 3. NF channel activation based on the DL-PFC+SC analysis with the BP 0.01- 0.09Hz filter in relation with, A. the gauge control feeling (Control vs. No Control), and B. the strategy used by the participants to control the gauge. Cheering strategies (e.g. self- encouragement); Concentration (e.g. being focused on something); Visualization strategies, (e.g. visual imagination); Anger (e.g. getting angry towards the gauge or himself/herself). Participants who had a positive gauge control feeling are in red while participants with no control feeling are in blue. The number of subjects for each condition are indicated in the pie charts. Chi-square comparison control vs. no control, **p* < 0.05.

Discussion

In the present study, 30 individuals performed a single session of fNIRS-based neurofeedback (NF) targeting the DL-PFC bilaterally that consisted of 15 trials of NF with 30-sec alternating resting periods.

For the BP 0.01-0.09 Hz filter without SC regression among the 10 of 15 best trials, 18 out of 30 participants (60%) had at least one channel of interest significantly activated during NF. This result is in line with the literature, where 15 to 30 % of participants are categorized as non-responders to NF [1,32]. In accordance with Munoz-Mendes’ taxonomy, our NF paradigm involved an “active overt uncued task” [33]. Indeed, the rewarding element was explicitly presented on a computer screen and participants were aware of its meaning (the gauge level was directly linked to the degree of their bilateral DL-PFC activity) but no explicit strategy was advised [33]. Based on the operant conditioning theory [1,31], individuals controlled the gauge via a series of trials and errors using their own mental strategies to find the best one. This may explain inconsistencies across NF trials as some participants tried different mental strategies across the session.

We found that the BP 0.01-0.09 Hz filter, as previously suggested [18] was better suited than the BP 0.01-0.2 Hz filter to specifically measure brain activation restricted to NF channels across the DL-PFC. Given that NF might promote intermittent neuronal activation, depending on participant’s success, a wider high pass filter might not be well-suited when neural activation is not evoked. However, current recommendations suggest using a low-pass filter of 0.5 Hz, as it will not remove too much signal related to brain activation [19].

When considering short-channel (SC) regression, we found that 13 individuals significantly activated at least one channel among four covering the DL-PFC. However, these results also highlight that 8 individuals had a false positive signal, where non-cortical increased HbO2 contributed to increased NF signal, whereas three individuals had a false negative signal, as SC regression revealed that at least one channel of interest was activated. These results suggest the importance of accounting for extra-cerebral physiological signals that could bias the NF signal retransmitted to the participants. Indeed, some individuals may have been reinforced towards a mental strategy that was based upon non-cerebral signal. Conversely, others may have abandoned a potentially good strategy that might have been influenced by non-cerebral signal. To address this issue, it could be useful to implement real-time SC regression during NF. An anti-correlation method, based on the assumption that changes in HbO2 and HbR are anti-correlated, could be used as a surrogate approach. However, since HbO2 and HbR are not always anti-correlated, a regression based on this approach could lead to errors [44].

With SC regression and within the 13 individuals for whom we detected at least one activated NF channel, 8 had increased activity in other frontal regions and 4 in the motor region. As we provided non-directive instructions and only suggested to engage mental strategies related to cognitive control, some mental strategies used by the participants may have recruited brain areas beyond the DL-PFC. For instance, with SC regression, 4 participants out of the 13 showed an activation within the motor cortex. This brain area may have been recruited in relation with the type of mental strategy employed, such as visualization leading to movement picturing. However, the group of participants with subjective feelings of controlling the gauge demonstrated no activated channels in the motor cortex.

Interestingly, we detected activation in the 12 channels covering the frontal brain region, independently ofdetected activation in the NF channels. In an fMRI- based NF meta-analysis of healthy individuals, it has been shown that NF training involves the recruitment of many other brain regions beyond the targeted brain region or networks [45]. These regions included the anterior insula, basal ganglia, posterior anterior cingulate cortex, ventro-lateral prefrontal cortex, DL-PFC, premotor cortex, a large cluster in parieto-temporal region and temporo-occipital junction. Thus, NF, independently of the target brain structure, requires or concomitantly induces the activation of brain regions involved in self-regulation and cognitive control, as well as motor and visual regions. This supports the idea that neural mechanisms underlying learning requires cognitive regulation and control, in turn explaining the widespread PFC activation of some participants in our study.

Gaume et al. (2016) proposed a general model of biofeedback learning, which involves the generalization of skill learning to higher level cognitive functioning, and can be applied to NF. Thus, participants perform tasks which elicit brain networks involved in executive functioning, self-control and error detection. Learning also requires the recruitment of brain areas involved in working memory. If the NF paradigm involves a motivating condition (essentially related to voluntary and explicit biofeedback, i.e., the participant wants to succeed in exerting control over the variable), then the reward system is activated. Finally, learning results in the formation of integrated neural rearrangements through the strengthening of synapses in regions involved in memory. Once learning is integrated there will be less cognitive involvement in coordinating the neural rearrangements [30]. In line with our results, Gaume’s theory and Emmert’s meta-analysis described above support the value of repeated sessions to let individuals identify the most effective mental strategies and to consolidate learning processes. Future work should consider examining whether repeated DL-PFC-targeted NF sessions lead to a decrease in other frontal activations or is it specific to DL-PFC activation, reflecting a learning success.

The association between the subjective feeling of control of the visual gauge elevation is our objective success criterion (i.e. one channel of interest activated with the DL-PFC+SC analysis). There, we found that 9 out of 15 participants who reported a feeling of controlling the gauge were accurate in their self-evaluation. On the contrary, 4 out of 15 participants in the group of participants with no control feeling had at least one channel of interest activated. This suggests that participants experiencing the positive feeling of controlling the gauge tended to better evaluate their ability to perform the NF task compared to others. In addition, when considering the gender of the participants, it appeared that women were more accurate in subjective perceptions than men.Six of 8 women had at least one activated NF channel and a control feeling, whereas nearly half of the men reported no control while there was an activation. Individuals had a relatively accurate perception of control relative to NF channels activation, especially when considering that only a single session of NF was performed with no basal reference.

Concerning the strategy used, the cheering strategy appeared to be more effective in men, although no significant difference was observed between men and women. This result is in line with a previous study that assessed through a single EEG-based NF session an upregulation of the sensorimotor rhythm, where no preferential strategy for efficient NF was reported [46]. In our study, the proportion of mental strategies within groups was not indicative of NF success. Indeed, a single NF session may have led the participant to explore different strategy approaches rather than reinforce a singleeffective strategy. In addition, some of our participants tried 2 or 3 different strategies (even in the same category), which may be counterproductive and lead to overloaded cognitive resources [34]. In a fNIRS-based NF training protocol targeting the PFC [47], through repeated sessions, the applied strategy “mental to-do-list” was associated with increased activity but only in 5 out of 13 participants. There is consequently no consensus in the literature regarding a preferential effective strategy related to NF success, reinforcing the idea that letting individuals find their personal best strategy is preferable.

Conclusions

Here, we demonstrated that a single session of 15 repeated NF trials targeting the DL-PFC promoted a significant activation in at least one of four NF channels covering the DL-PFC for 13 participants among 30. This was after considering the 10 best NF trials and accounting for physiological noise generated from the scalp. The current study should be considered in the light of its strengths and weaknesses. As we did not provide explicit instruction related to a specific mental strategy, 15 trials may have limited the possible strategies that participants could explore. More NF trials or sessions may be required to do so more accurately. Moreover, adding a control group in a double-blind paradigm would increase statistical robustness, and avoid experimental biases [48,49]. Indeed, NF training without a control condition contributes to the inability to establish causality, such as to demonstrate neurophysiological specificity of the NF training, to exclude placebo, global (spatially non-specific) and behavioral effects [49]. Nevertheless, this kind of experimental paradigm is relevant in early stages of methodological developments (before clinical trial, e.g. pilot studies), especially to investigate whether individuals are able to control the ROI or not. Beyond these drawbacks, we point out that the implementation of SC regression for real-time NF signal computation might be well suited to avoid non-cerebral signal influence. This might have importance when the NF mental strategy is examined.

Supporting information

Supplementary Figure 1. Block average of the four NF channels in the DL-PFC, for NF and rest periods and for each individual without short channels (SC) regression and with A. BP 0.01-0.09Hz filter and B. BP 0.01-0.2Hz filter. Individuals are separated in two groups, depending on their feeling of control of the gauge (n=15 for each group). Individuals who had at least one channel of interest significantly activated during NF are surrounded by a full rectangle, whereas individuals who had no channel of interest significantly activated are surrounded by a dotted rectangle.

Supplementary Table 1. Mental strategies. The mental strategies were acquired by verbal report (interview) at the end of the neurofeedback session in front of two experimenters. These reports were anonymized and then the strategies were classified by the experimenters and approved by another experimenter who was not present during the neurofeedback sessions. Strategies reported as "Cheering" are related to self-encouragement and/or gauge encouragement. Visualization strategies are related to visualizing a specific situation or object (including memory recall). Concentration strategies are related to any strategy that requires specific mental effort beyond visualization or memory recall. Emotion strategies are related to any strategy that was specifically focused on an internal feeling. Because some participants tried different strategies, the strategy that was most applied and/or related to their internal sense of control over the gauge was selected for analysis. Indeed, when a strategy seemed to be more effective for controlling the gauge, it was the strategy most used by the participant.

Supplementary Table 2. CREDlist.
