# Supplementary material for: Subjective feeling of control during fNIRS-based neurofeedback targeting the DL-PFC is related to neural activation determined with short-channel correction
Source: PLoS One. 2023 Aug 16;18(8):e0290005. doi: 10.1371/journal.pone.0290005 (PMC10431651; doi:10.1371/journal.pone.0290005)
Supplement: Attachment — Submitted filename: Response_to_reviewer.docx [file pone.0290005.s008.docx]

**Reviewer #1**: Great work on this revision! I appreciate your time and energy in making this paper more widely understood to less expert audiences and for improving the level of technical detail and specificity for replication purposes. There are a few very minor grammar and typo edits to be made and I think this manuscript will be ready for publication depending on the what the editors believes is best.

**Answer**: We would like to thank you for pointing out persistent grammatical errors, which have been corrected according to your suggestions. We have also added the year of reference 18 (Pinti, 2019) and corrected a few errors in the citation style of references.
